# Hunters’ acceptability of the surveillance system and alternative surveillance strategies for classical swine fever in wild boar - a participatory approach

**DOI:** 10.1186/s12917-016-0822-5

**Published:** 2016-09-06

**Authors:** Katja Schulz, Clémentine Calba, Marisa Peyre, Christoph Staubach, Franz J. Conraths

**Affiliations:** 1 Friedrich-Loeffler-Institut, Federal Research Institute for Animal Health, Institute of Epidemiology, Südufer 10, 17493 Greifswald, Insel Riems Germany; 2 Centre de Coopération Internationale en Recherche Agronomique Pour le Développement (CIRAD), Département ES, UPR AGIRs, TA C22/E, Campus International de Baillarguet, 34398 Montpellier Cedex 5, France

**Keywords:** Classical swine fever, Surveillance, Participatory epidemiology, Acceptability, Wild boar

## Abstract

**Background:**

Surveillance measures can only be effective if key players in the system accept them. Acceptability, which describes the willingness of persons to contribute, is often analyzed using participatory methods. Participatory epidemiology enables the active involvement of key players in the assessment of epidemiological issues. In the present study, we used a participatory method recently developed by CIRAD (Centre de Coopération Internationale en Recherche Agronomique pour le Développement) to evaluate the functionality and acceptability of Classical Swine Fever (CSF) surveillance in wild boar in Germany, which is highly dependent on the participation of hunters. The acceptability of alternative surveillance strategies was also analyzed. By conducting focus group discussions, potential vulnerabilities in the system were detected and feasible alternative surveillance strategies identified.

**Results:**

Trust in the current surveillance system is high, whereas the acceptability of the operation of the system is medium. Analysis of the acceptability of alternative surveillance strategies showed how risk-based surveillance approaches can be combined to develop strategies that have sufficient support and functionality. Furthermore, some surveillance strategies were clearly rejected by the hunters. Thus, the implementation of such strategies may be difficult.

**Conclusions:**

Participatory methods can be used to evaluate the functionality and acceptability of existing surveillance plans for CSF among hunters and to optimize plans regarding their chances of successful implementation.

## Background

The emergence of Classical Swine Fever (CSF) within a wild boar population is a highly undesirable event. This is mainly due to the potential transmissibility of the infectious agent from wild boar into domestic pig populations [1–3]. Also, strict movement regulations affecting domestic pig holdings are implemented for the holdings in the restriction zone around a CSF outbreak according to EU and national legislation.

CSF is caused by an enveloped single-stranded RNA pestivirus, which belongs to the family of *Flaviviridae* [4]. The course of disease is determined by several factors such as viral virulence, the age of the infected animal and its immune status [5]. Both the acute and chronic forms of disease cause non-specific clinical signs including fever, inappetence and weakness resulting in increased mortality in the affected population [5–7]. Consequently, introduction of the virus into commercial pig holdings usually entails huge economic consequences [8, 9].

Since the first occurrence of the CSF-virus in 1833 in the United States, the disease has spread through the American and European continents [10]. In the 1970s, Germany had several years without any detected CSF-cases within the wild boar population. However, from the beginning of the 1980s until the most recent cases in 2009, the disease has occurred in a number of German federal states [1, 11–13]. Since 2012, Germany has been recognized as officially free from CSF [13]. To demonstrate freedom from CSF on a regular basis, which is prerequisite for participating in international trade, Germany has to implement regular surveillance for the disease in the wild boar population which fulfils the requirements of the current legislation in the European Union (Commission Decision 2002/106/EG).

Private hunters typically conduct sampling of harvested wild boar in Germany. However, in times of freedom from disease this happens on a voluntary basis, since hunters are often private persons and there is no legal requirement based on which hunters can be forced to support surveillance activities in times of disease freedom. In a few federal states, they are compensated for their financial expenses and their time [14]. However, neither in Rhineland-Palatinate nor in Mecklenburg-Western Pomerania, from where hunters were recruited in the present study, compensations are paid. Many surveillance activities for CSF thus rely on the involvement of hunters, in particular in relation to sampling shot wild boar. It is therefore essential that hunters accept the necessity of these surveillance activities and contribute to their implementation. Their front-line role in the system makes it equally important to consider their concerns during the development of new or the modification of existing surveillance strategies.

Meynard et al. [15] defined acceptance as the willingness of a person or an institution to participate in the implementation of a surveillance system. By evaluating acceptability, one can identify clues for improving the operation of the system [16]. Acceptance is often measured using participatory methods [17, 18].

Participatory epidemiology originates from the fields of social sciences and public health [19]. It comprises of a new range of methods and tools, which can also be used in the field of veterinary epidemiology. So far, participatory epidemiology has been mainly used in developing countries but it has been recently applied in the European Union (EU) to develop a method to assess the acceptability of surveillance systems (AccEPT) [20–23]. Its principle is based on the active participation of concerned parties in problem-solving, process-optimization and the development of new concepts. Visualization tools are used to facilitate discussions between involved persons or stakeholders [23]. Tools such as proportional piling, where categories can be formed and scored, make it possible to evaluate outcomes not only qualitatively but also quantitatively. By including participatory epidemiology in the design of new or the evaluation of existing surveillance strategies, there is a chance to integrate surveillance actors’ knowledge or experience, which could otherwise remain inaccessible (23). Moreover, participatory epidemiology can help to prevent surveillance from failing due to lack of acceptability by key players. Since a number of studies have shown that the application of participatory methods could improve the outcome of animal health surveillance in developing countries, it is hypothesized that the use of these methods could be beneficial to industrialized countries [23–26].

The aim of the study was to evaluate the acceptability of the currently implemented surveillance strategy for CSF in wild boar using the AccEPT method (23). Additionally, alternatives to the conventional strategy were constructed on the basis of risk-factors for a higher probability of CSF virus infection and detection in wild boar in Germany. The acceptability of these strategies by hunters was also evaluated.

## Methods

### Recruitment of hunters

From April 2015 to June 2015, hunters were recruited from districts of the federal states of Mecklenburg-Western Pomerania (MV) and Rhineland-Palatinate (RP) by contacting the responsible hunting authorities at the district level (Landkreis) via email and contacts with the so called “Kreisjagdmeister” were established. The Kreisjagdmeister is elected by the hunters of a district and represents the hunters in the communication with the district hunting authority. With the help of the Kreisjagdmeister, hunters were contacted and meetings arranged with those who were willing to participate. It was planned to recruit 3–5 hunters, who formed a focus group, per participating district.

Five groups of hunters were interviewed in RP and three in MV. The group discussions took place between the 16 June and the 7 July 2015. On average, the meetings lasted approximately two hours. The biggest group consisted of seven hunters whereas only two hunters took part in the smallest discussion group. In total, 27 male hunters and one female hunter participated. For the majority of participants, hunting was a hobby. All hunters stated that they hunted several times a week. The estimated average age was above 50 years; most hunters had thus been actively involved, when CSF outbreaks occurred in the respective federal states.

### Surveillance system of CSF in wild boar

To illustrate the processes and the responsibilities within the surveillance system for CSF in wild boar, an information flow diagram was developed. This was done by interviewing experts and by using information from the German contingency plan for CSF (Tierseuchenbekämpfungshandbuch) [27].

**Fig. 1:**
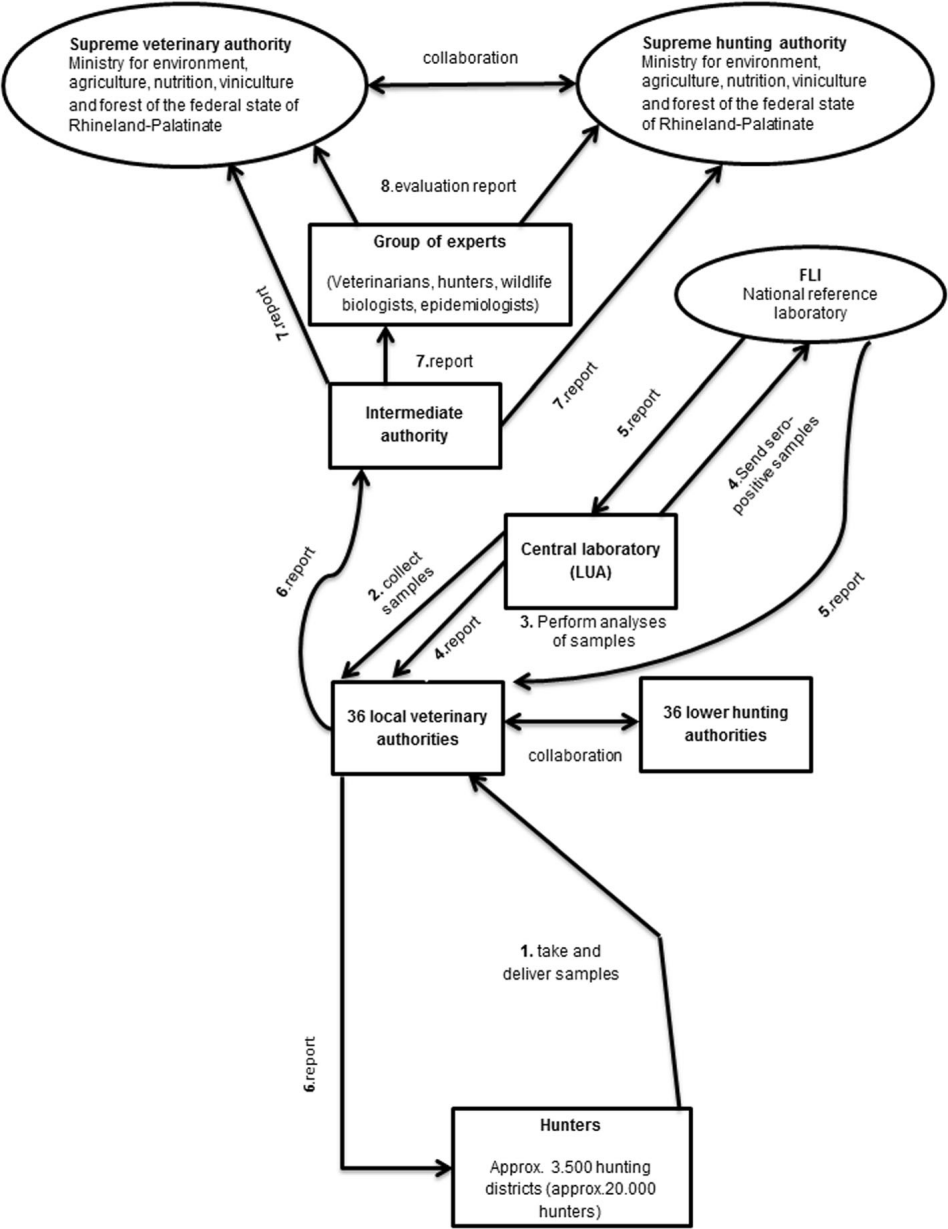
Information flow (*bottom up*) within the surveillance system of the currently implemented, active surveillance for Classical Swine Fever in wild boar in times of disease freedom on the basis of the federal state of Rhineland-Palatinate

By creating a diagram of the information flow within the surveillance system for CSF in wild boar, it became obvious that hunters play a key role in this system (Fig. 1). In times of freedom from disease, hunters take samples from wild boar and deliver the samples to the veterinary authority. The central state laboratory (‘Landesuntersuchungsamt, LUA) plays another key role. It collects and analyses samples from the 36 local district veterinary authorities. Negative results are reported back to the local veterinary authorities, whereas samples that tested inconclusive or positive are sent to the Friedrich-Loeffler-Institute (FLI) for further investigation. The FLI reports results back to the LUA and simultaneously to the local veterinary authorities. Subsequently, the local veterinary authorities report the results to the intermediate authority, which is an interposed authority between the authorities of the district and the supreme authority, and back to the hunters. The intermediate authority reports the results to a group of experts, the supreme veterinary and the supreme hunting authority of the federal state. Finally, the group of experts evaluates the surveillance results and reports these evaluations back to the supreme veterinary and hunting authority of the federal state (Fig. 1).

To avoid any biased responses, the generated information flow diagram was not presented to the hunters.

### Implementation of participatory methods

Focus group discussions were conducted. The meetings were performed always by the same scientist and recorded with an Olympus dictation machine with the participants’ consent.

#### Acceptability of the current surveillance system

The method used was adapted from the AccEPT method recently developed [23]. Where not stated differently, analyses of the discussions were done by qualitative as well as semi-quantitative analyses by using the scoring system of [23].

For further analysis, the results of the group discussions of hunters of each federal state were first summarized. For the final evaluation, all group discussions were analyzed together.

The investigation of the acceptability of the surveillance system was divided into three parts:

After the implementations of each part, the hunters were asked to explain their choices and motivations. These discussions were part of the descriptive analyses.

1. Evaluation of the **acceptability of the operation** of the system.
  These investigations were subdivided into:
  - Satisfaction of the hunters with their own role in the surveillance system.
    An information flow diagram was created by the hunters. The hunters were asked to state, which institution they would contact in case of a suspicious wild boar and what they thought would happen with this information. The analysis of the satisfaction of the hunters with their own role was done through a semi-quantitative analysis of the discussions during the creation of the flow diagram as well as through a merely qualitative analysis of the discussions. The scoring system of the semi-quantitative analysis is described in detail in the study of [23].
  - Satisfaction of the hunters with their social relationships within the hunting network.
    A relational diagram was used to identify the persons or institutions the hunters were in contact with regarding their hunting activities. The hunters were asked to name persons or institutions within their hunting network. In addition to [23], we used smileys to visualize the satisfaction with the named persons/institutions. After reaching an agreement, the groups were asked to put one smiley to each of the listed persons/institutions. By assigning scores to each smiley used, satisfaction could be analyzed in a semi-quantitative way (Table 1).

**Table 1:**
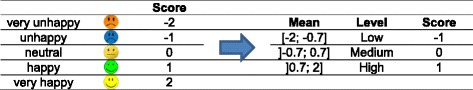
Assessment of the satisfaction of hunters with the relationships within the hunting network
  - Consequences for the hunters in the case of a suspicion of CSF in wild boar.
    By creating an impact diagram as described in the study of [23], the hunters were asked to list consequences they were expecting for themselves in the case of a CSF outbreak in wild boar. They were motivated to distinguish between negative and positive consequences. Afterwards, they were asked to explain their choice. To score the named consequences, proportional piling was implemented. The method and also the analyses are described in detail in [23].
    The scores of parts a–c were summed up and arithmetic means calculated to determine the acceptability of the operation of the system by the hunters (Table 2).

**Table 2:** Assessment of the level of acceptability of the operation of the surveillance system

Level	Score
Low	[−1; −0.33]
Medium	]−0.33; 0.33]
High	] 0.33; 1]
2. Evaluation of **acceptability of the objective** of the surveillance system.
  The hunters were asked to describe to the best of their knowledge the information flow to be observed if they find a dead wild boar or an animal-suspected being CSF-infected. The acceptability of the objective of the surveillance system, which is demonstrating freedom from disease, was investigated by asking the hunters directly after the creation of the information flow diagram. It was asked, what, in their opinion, is the objective of the currently in Germany implemented surveillance system for CSF in wild boar. The results of the discussions were analyzed in a semi-quantitative way using the scoring system described by [23].
3. Evaluation of the trust in the surveillance system
  The trust of the hunters in the functionality of the surveillance system of CSF in wild boar was assessed by using proportional piling [23] after creation the flow diagram. One hundred small tokens were presented to the hunters and they were asked to pile these in relation to their trust either in a field for “Trust” or in another field for “Distrust”. It was asked, whether the hunters believed that different key players in the system and their work are expedient and reliable and if the system works effectively. After they had allocated the tokens to the two fields, the hunters were asked to pile the stones of the field “Trust” proportional to their trust into the different persons within the flow diagram.

#### Acceptability of alternative surveillance systems

Due to time restrictions and in the interest of maintaining a good working relationship with the hunters, only a manageable number of alternative surveillance strategies could be presented.

Alternative strategy 1 (Table 3) was presented assuming that there is an increased probability of detecting CSF in animals identified through passive surveillance. Infected animals are more likely to be involved in road traffic accidents and an increased mortality of infected animals has been demonstrated, particularly in the beginning of an outbreak [6, 28, 29]. The acceptability of alternative strategy 2 was examined as it can be expected that sampling in only 4 months of the year would result in less cost and work load. Alternative strategy 3 was presented to the hunters because the probability of detecting CSF-specific antibodies in older animals is higher due to the cumulatively increased seroprevalence in the boar population [30–32]. In the alternative strategy 4, active and passive sampling elements were combined. As it is recommended in Commission Decision 2002/106/EG to examine also all animals found through passive surveillance, it was important to establish if a surveillance strategy consisting of active and passive elements would be accepted by the hunters.

**Table 3:** Chosen strategies for CSF surveillance in wild boar for which the acceptability of hunters were evaluated

1. Currently implemented (strategy 1) 59 samples of the whole hunting bag per district within one year
2. Passive (alternative strategy 1) Sampling of wild boar found dead, shot sick or involved in a road traffic accident
3. Quarterly (alternative strategy 2) 59 samples of the whole hunting bag per district within one year; sampling only quarterly e.g. January, April, July, October
4. Sub-adults (alternative strategy 3) 59 samples, only from sub-adults, of the whole hunting bag per district within one year
5. Strategy 1 combined with 50 % passive (alternative strategy 4) Sampling 50 % of all wild boar which were found dead, shot sick or were involved in a road traffic accident plus 59 samples of the whole hunting bag per district within one year

The method used to evaluate the hunters’ acceptability of the conventional surveillance strategy and of the alternative surveillance strategies for CSF in wild boar (*A*_*s*_, *s* ∈ {strategy 1, alternative strategy 1, alternative strategy 2, alternative strategy 3 and alternative strategy 4}) (Table 3), was newly developed and is described in detail in the following section.

Five strategies were presented to the hunters (Table 3). Each hunter got five smileys, i.e. one smiley for each level of satisfaction (*m* =5) (Table 1). The hunters were asked to distribute these smileys on the presented strategies depending on their level of acceptance. For each strategy, the arithmetic mean was calculated according to

$$ {A}_s = \frac{{\displaystyle {\sum}_{i=1}^m}{x}_i\cdot s{c}_i}{n} $$

where *i* = 1 represents the level of satisfaction, *x*_*i*_ the number of hunters giving score i (*sc*_*i*_) and *n* the total number of hunters. The scoring values *sc*_*i*_ for each smiley are shown in Table 1. All hunters were asked to state reasons for their choice. The discussions were analyzed descriptively.

## Results

### Acceptability of the current surveillance system

**Fig. 2:**
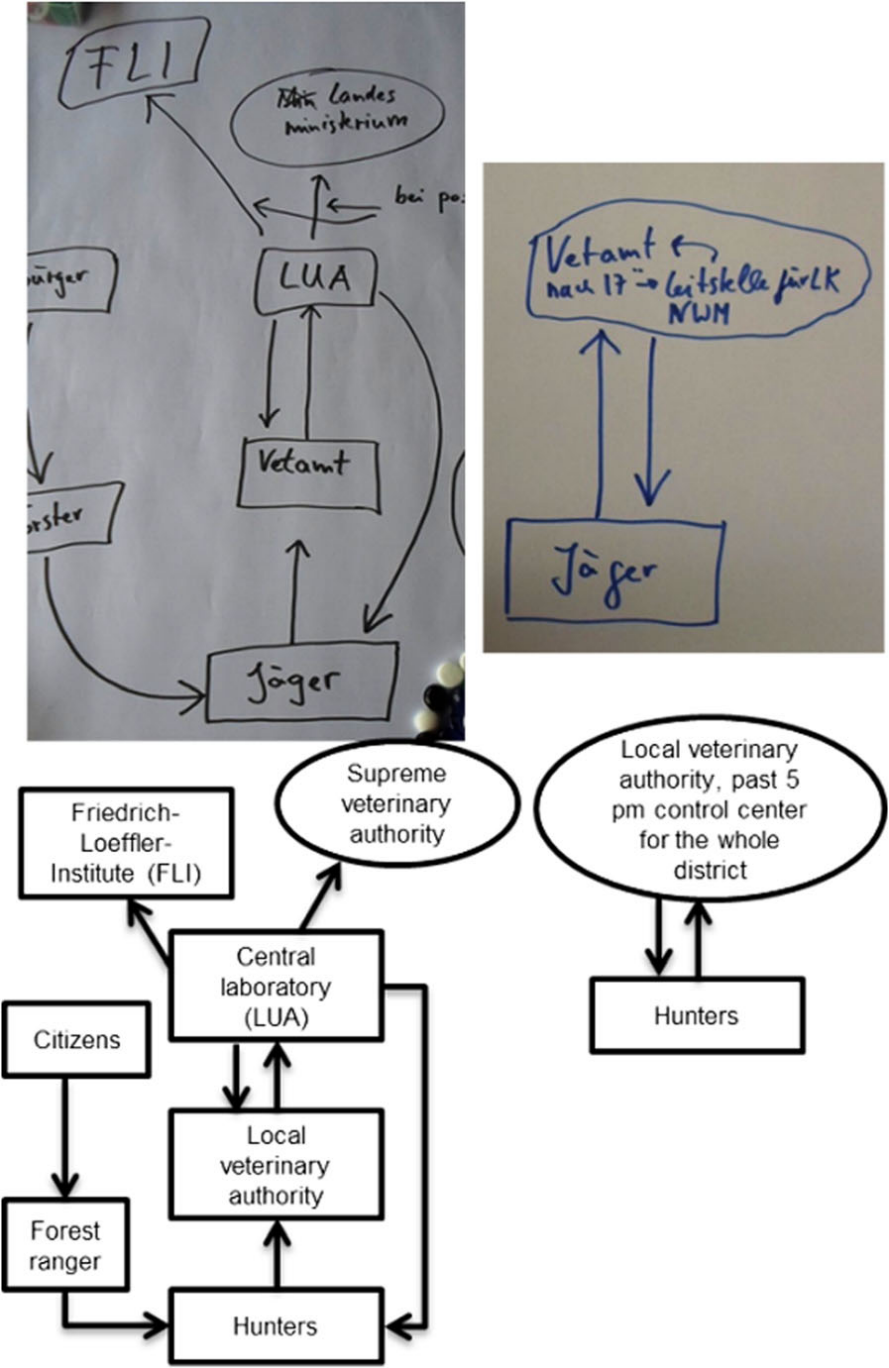
Two original examples of flow diagrams to evaluate the satisfaction of the hunters with their own role within the surveillance system for CSF in wild boar (English translation subjacent). Left: The group could illustrate the information flow very detailed. Right: The group could only identify the local veterinary authority as the closest institution

1. Acceptability of the operation
  - By creating a flow diagram, all groups could identify the local veterinary authorities as the institution that should be directly contacted in the case of suspicion of CSF in a wild boar. Knowledge about the processes above the veterinary authority differed between the groups (Fig. 2). Good cooperation with the closest institution was stated as a positive reflection of the status quo, whereas a lack of transparency regarding the processes within the supreme authorities was named as a negative aspect. During the preparation of the flow diagram, mainly positive discussions points were noted in half of the groups. For the others, positive and negative points were balanced. The scoring of the discussion points is described in [23]. The satisfaction of the hunters with their own role within the system resulted in a medium score of 0.45 (Table 4).

**Table 4:** Results of the calculations of the acceptability of the surveillance system of CSF by hunters

	Acceptability of the operation of the system			Trust in the system	Acceptability of the objective of the system
	satisfaction with their own role (RP 0.6) (MV 0.3) (Mean 0.45)	satisfaction with the relationships (RP 0) (MV 0.7) (Mean 0.35)	consequences in a suspicious case (RP–1) (MV–1) (Mean–1)		
RP		−0.1		0.8	−0.6
MV		0		0.7	0
All hunters		−0.1		0.8	−0.4

*Abbreviations*: *RP* indicates Rhineland-Palatinate, *MV* indicates Mecklenburg-Western Pomerania
  - By generating a relational diagram, a total of 29 persons or institutions were named by the different groups as those, with which they had contact within the network of hunting. The lower hunting authority was named by all eight groups. Forestry authorities, farming and veterinary authorities were mentioned by seven groups. Eleven persons or institutions including the media and meat consumers were listed by a single group. During the discussions, all groups emphasized that they found it difficult to make generally applicable statements concerning the quality of contacts as it was believed to be highly dependent on the individual correspondents. The relationship to authorities that were closed to the hunters, such as the lower hunting or the local veterinary authority was described as very good. By contrast, the relationship to farmers, forest officers or the public was regarded as difficult. Problems were mainly seen as a consequence of conflicts of interest.
  - During the development of the impact diagram, all hunters had difficulties naming a positive consequence for them after the discovery of a wild boar suspected of being infected with CSF. Two groups failed to list positive consequences. Named positive consequences included a learning effect and the reduction of the wild boar population due to CSF and its control. Among the negative consequences, it was mentioned by all eight groups, that a CSF-suspected boar resulted in higher workload, increased expenses and hunting restrictions.
    By summarizing these three parts for both study areas, a medium level of acceptability of the operation of the surveillance system was calculated with a score of −0.1 (Table 4, Fig. 3).

**Fig. 3:**
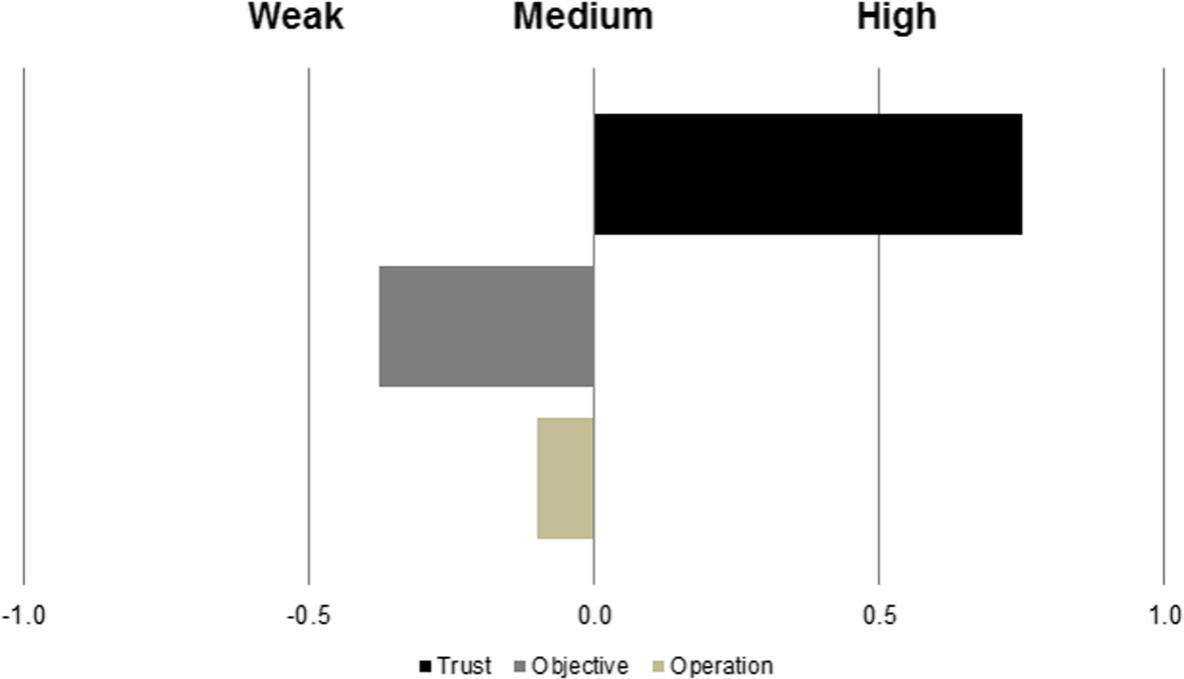
Level of acceptability of the objective and the operation of the CSF surveillance system as well as the level of trust in the surveillance system by hunters from Mecklenburg-Western Pomerania and Rhineland-Palatinate
2. Acceptability of the objective of the system
  No group could exactly define the objective “demonstrating freedom from disease”.
  Consequential, the calculated level for the acceptability of the objective of the surveillance system for CSF in wild boar (demonstrating freedom from disease) by all hunters was a score of −0.4 (Table 4, Fig. 3).
3. Evaluation of the trust in the surveillance system
  The trust in the surveillance system was high in all groups and resulted in a score of 0.8 (Table 4, Fig. 3). This was mainly justified by the argument that “past outbreaks showed that the system works” (focus group with hunters, 16.06.2015). The greatest trust was assigned to the authorities working close to the hunters like the lower hunting authority and the local veterinary authority. However, the trust in the hunters themselves was low, which was explained by the heterogeneity of the group of all hunters. It was mentioned that some hunters might ignore a dead wild boar for convenience or due to lack of time. Also, due to lack of transparency, the trust in the supreme authorities of the respective federal states was regarded as relatively low.

### Acceptability of alternative surveillance systems

**Fig. 4:**
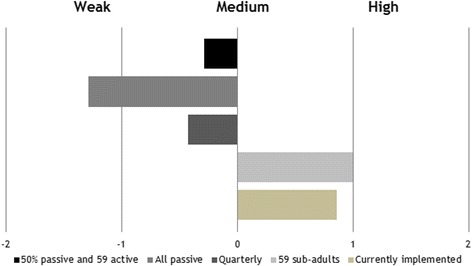
Level of acceptability of different CSF surveillance strategies by all hunters of Rhineland-Palatinate and Mecklenburg-Western Pomerania

Summarizing the results and the discussions in the eight groups of hunters, the currently implemented strategy (strategy 1) was well accepted and resulted in a score of 0.9. Alternative strategy 1 (Table 3) showed the lowest level of acceptability with a score of −1.3 (Fig. 4). The hunters stated that dead animals are very often not found. Additionally they avoid sampling dead animals due to disgust and the effort, which is unprofitable to them. Alternative strategy 3 resulted in the best score of 1 (Fig. 4), also because sub-adults represent the group from which animals are shot most often anyway. Alternative strategies 2 and 4 were moderately accepted. The former scored −0.4 (Fig. 4); quarterly sampling was criticized as “too complicated” (focus groups with hunters, 16.06. and 17.06.2015) and the hunters emphasized that shooting wild boar is not predictable, especially in the summer months. Similarly, the latter alternative received a score of −0.3, which was mainly due to the passive element of the strategy.

## Discussion

Participatory methods are still rarely used in the field of veterinary epidemiology, particularly in industrialized countries [23, 33]. This is, among other reasons, due to difficulties in interpreting results since the analyses have often to be done in a qualitative way. The interpretation of discussions brings the danger of subjectivity, which is why it is recommended for more than one scientist to conduct the studies. In the present study, only one scientist could hold the meetings due to time and cost restrictions. However, for transparency, all discussions were recorded.

Only hunters from two federal states (MV and RP) were recruited. Both federal states have been affected by CSF in the past and most of the recruited hunters were estimated to be older than 50 years and practiced hunting in times of CSF outbreaks [13]. Thus, it cannot be assumed that the experience and knowledge of this group regarding CSF is representative of the national average for all hunters. Presumably, experience and knowledge regarding CSF in wild boar were above average. To avoid these inherent bias factors, it would be necessary to disperse the selection of hunters more widely. However, the number of interviews in this study was limited due to restricted resources.

A cause of bias in all participatory studies is the method of participant selection [23]. By contacting the lower hunting authority, it is likely that we reached hunters who were in contact with the authorities anyway. These hunters are more likely to be interested and to cooperate with the authorities.

This study included only a small sample size. Only 28 of approximately 400,000 registered German hunters participated (https://www.jagdverband.de/content/jagdscheininhaber-deutschland). Qualitative studies aim to reach “theoretical saturation” meaning that no new information can be obtained from interviewees [34]. Although this theory was not implemented consciously, no new information was added after the third group discussion, which may indicate that theoretical saturation had already been reached.

In spite of the listed limitations, our study clearly demonstrates that the acceptability of the current CSF surveillance system in German wild boars is only rated as medium by hunters, a major stakeholder group within the surveillance program.

Major determinants of this score were that the knowledge of the procedure in the event of discovering a wild boar suspected of CSF was excellent and that trust in these immediate contacts was high. However, the hunters could not define the overarching objective of the program and lacked trust in the transparency of operations at the supreme level of each federal state. Furthermore, some hunters could not identify even a single positive outcome for reporting a CSF-suspicious carcass; this is clearly problematic for a scheme run on a voluntary basis. These findings correlate with the hypothesis of [26] that communication between stakeholders and executing key players needs to be improved. Better communication and more information from the supreme authorities could help to increase the acceptability of the system and thereby the functionality.

The analysis of the acceptability of the different surveillance strategies showed that there is a need to improve the willingness to sample animals resulting from passive surveillance since it is known that the probability of detecting CSF in these animals is higher [28, 29]. To sample quarterly (alternative strategy 2) was not well accepted by the hunters. It is assumed that this strategy would be cost-effective as transportation and sample handling are more concentrated in time. However, according to the hunters, it is impractical due to the seasonal variation of the numbers of hunted boar. These findings strongly emphasize the advantages of participatory methods. Even if a surveillance strategy shows a theoretical advantage such as a high detection probability or low costs, it will be hard to apply when hunters are not willing or capable of supporting it because they have strong arguments against a certain strategy. The high acceptability for sampling only in the age-class of sub-adult animals demonstrates the additional potential for combining risk-based surveillance strategies with participatory methods.

## Conclusions

The acceptability among German hunters regarding a surveillance system for CSF in wild boar was evaluated by participatory methods recently adapted to evaluation of surveillance systems. The methods were well accepted by all interviewed groups of hunters. It was shown that acceptability is an attribute that can be used to gain important knowledge and to take experience of key players into account, which is often overlooked in the planning of surveillance strategies. Therefore, participatory epidemiology can be considered as an additional tool to inform decision-making on the improvement of surveillance strategies to inform disease freedom status.

## Abbreviations

CSF: Classical Swine Fever
FLI: Friedrich-Loeffler-Institute
LUA: Central Laboratory of Rhineland-Palatinate
MV: Mecklenburg-Western Pomerania
RP: Rhineland-Palatinate
